# Robust multi-site MR data processing: iterative optimization of bias correction, tissue classification, and registration

**DOI:** 10.3389/fninf.2013.00029

**Published:** 2013-11-18

**Authors:** Eun Young Kim, Hans J. Johnson

**Affiliations:** ^1^Biomedical Engineering Department, University of IowaIowa City, IA, USA; ^2^Psychiatry, University of Iowa Hospital and ClinicIowa City, IA, USA

**Keywords:** segmentation, registration, inhomogeneity correction, tissue classification

## Abstract

A robust multi-modal tool, for automated registration, bias correction, and tissue classification, has been implemented for large-scale heterogeneous multi-site longitudinal MR data analysis. This work focused on improving the an iterative optimization framework between bias-correction, registration, and tissue classification inspired from previous work. The primary contributions are robustness improvements from incorporation of following four elements: (1) utilize multi-modal and repeated scans, (2) incorporate high-deformable registration, (3) use extended set of tissue definitions, and (4) use of multi-modal aware intensity-context priors. The benefits of these enhancements were investigated by a series of experiments with both simulated brain data set (BrainWeb) and by applying to highly-heterogeneous data from a 32 site imaging study with quality assessments through the expert visual inspection. The implementation of this tool is tailored for, but not limited to, large-scale data processing with great data variation with a flexible interface. In this paper, we describe enhancements to a joint registration, bias correction, and the tissue classification, that improve the generalizability and robustness for processing multi-modal longitudinal MR scans collected at multi-sites. The tool was evaluated by using both simulated and simulated and human subject MRI images. With these enhancements, the results showed improved robustness for large-scale heterogeneous MRI processing.

## 1. Introduction

Accurate and robust analysis of brain MR imaging from multi-site, multi-modal and longitudinal studies is a difficult problem. A key research technique for advancing the understanding of the human brain is the analysis of large collections of MR images (Cocosco et al., 1997; ADNI, 2004; Marcus et al., 2007; INDI, 2010; IXI, 2013). The largest data sets are commonly amalgamations of data collected from similar, but independent, research studies. Successful development of a fully automated analysis framework can reduce both the operator time requirement and measurement variability for clinical trial applications (Zijdenbos et al., 2002). A primary challenge associated with automating the analysis of large-scale and multi-site studies is the development of techniques to address large variabilities in scan properties due to data collection on different scanner manufacturer and scanning environments. High-quality registration and bias-field correction techniques become essential to ensure interpretation consistency of large data-sets collected from different scanners or multi-site studies. As a result, there has been an increased emphasis on automated tool development for multi-site MR image analysis.

Iterative optimization approaches have been proposed to achieve robust MR processing, and these often involve three main techniques: bias-field correction, registration, and tissue classification. Approaches that incorporate these three techniques are attractive because they recognize that these are naturally one interrelated and interconnected optimization problem. The improved intensity uniformity provided by bias-field correction produces better registration accuracy, and also enhances tissue classification. Correct tissue type identification helps to improve bias-field estimation, which in turn improves registration accuracy. In previous studies, Wells et al. (1996) proposed an adaptive segmentation method by using Expectation Maximization (EM) algorithm with simultaneous bias field correction. This idea was further advanced by work in Van Leemput et al. (1999); Prastawa et al. (2004). Each of these papers described an iterative method that alternates between bias correction and tissue classification within an EM algorithm framework. These previous works have been applied successfully in several research projects (Prastawa et al., 2003, 2004, 2005; Prastawa, 2007). The reported instances of these implementations, however, have only been applied with limited conditions: single-site, single-scanner, single-modality, affine registration and limited 3-tissue model.

In this paper, we expand upon the previously introduced procedure in Prastawa et al. (2004), and describe the algorithmic enhancements for increasing the robustness of the framework to be applicable to a large-scale multi-site heterogeneous data analysis (32 sites, 3000+ scan sessions). The improvements for robustness were achieved by incorporating four components: (1) a collective set of inputs including multi-modal and repeated scans to maximize utilization of data at hands, (2) a high-deformable registration approach for better correspondence to a subject from a atlas, (3) expanded tissue spatial prior definitions to 12 discrete tissue types (and 5 nuisance tissue types), and (4) intensity-constrained tissue priors, so called *region-specific intensity context priors*, based on *a priori* tissue-specific robust statistics. To evaluate our proposed method, we discuss the relative benefits of high deformable compared against affine registration and of the extended definition of tissue priors against traditional three tissue priors towards tissue classification accuracy of large-scale data (Section 3.1). In addition, we also provide visual inspection outcome and sample results of our tissue classification results compared with other well-established works in the field (Section 3.2). Finally, we conclude with a discussion of limitation and some possible future directions of development (Section 4).

## 2. Methods

**Figure 1:**
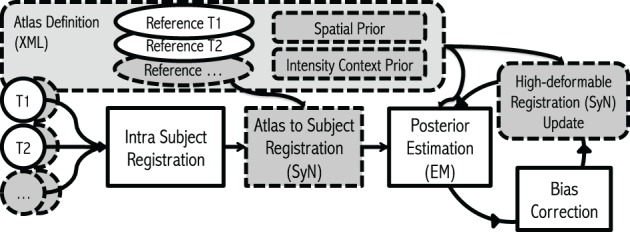
**Flowchart**. The framework takes any number of modalities, with any number of repetitions of scans as inputs. The algorithm starts with *Intra Subject Registration* to align all intra-session scans into first scan given. Then the initial *Atlas to Subject Registration* is performed to place all the atlas priors into subject space. Finally, the iterative process for *Posterior-Estimation*, *Bias Correction*, and *Registration Update* is repeated multiple times. Gray dashed boxes represent where our enhancements.

The implementation of our iterative framework incorporates (1) bias-field correction, (2) tissue classification, and (3) image registration with specific enhancements for robust processing of large-scale multi-site MR data. The basic philosophy of the framework has conceptual similarities to the works from Wells et al. (1996), Prastawa et al. (2004) and Avants et al. (2011a) with enhancements (dashed boxes in the Figure 1) that we have found useful for automated processing of large heterogeneous data.

*EM Algorithm for Bias Correction:* A core implementation of this work uses a general expectation-maximization (EM) algorithm (Wells et al., 1996; Van Leemput et al., 1999; Gelman et al., 2004; Avants et al., 2011a) by iterating distributional parameter estimation and individual voxel *y* classification at location *i* into *K* tissue types. The process assumes Gaussian mixture model *y_i_* ~ *N*(θ_*i*_) where θ_*i*_ = {μ_*i*_, σ_*i*_} with mean μ and variance σ^2^ of each tissue label Γ ∈ {*l*|*l* = 1 … *K*}. First step is the expectation (E) step to determine the expected posterior density function *p*(*y*_*i*_ | θ, Φ_*i*_) with estimated bias-field Φ_*i*_ (Van Leemput et al., 1999). Formulations in this paper are adapted from the works (Wells et al., 1996; Van Leemput et al., 1999; Gelman et al., 2004; Avants et al., 2011a).

*E-Step*

(1) $$p(y_{i}|\theta ,\Phi_{i})=\sum_{l}p\left(y_{i}|\Gamma_{i}=l,\theta_{l},\Phi_{i}\right)p(\Gamma_{i}=l),$$

with *p*(*y*_*i*_|Γ_*i*_ = *l*, θ_*l*_, Φ_*i*_) = *N*_σ,*l*_(*y*_*i*_ − μ_*l*_ − Φ_*i*_) and

(2) $$p\left(\Gamma_{i}=l\right)=\frac{t_{il}}{\sum_{l=1}^{k}t_{il}},\text{where }t_{il}\text{ is a tissue specific prior}$$

Then the maximization (M) step computes parameters of Gaussian θ and bias-field Φ by maximum likelihood estimation from current density function.

*M-Step*

(3) $$\begin{array}{l} \mu_{l}=\frac{\sum_{i}p\left(\Gamma_{i}=l|y_{i},\theta ,\Phi_{i}\right)\left(y_{i}-\Phi_{i}\right)}{\sum_{i}p\left(\Gamma_{i}=l|y_{i},\theta ,\Phi_{i}\right)},\\ \sigma_{l}^{2}=\frac{\sum_{i}p\text{​}\left(\Gamma_{i}=l|y_{i},\theta ,\Phi_{i}\right){\left(y_{i}-\mu_{l}-\Phi_{i}\right)}^{2}}{\sum_{i}p\text{​}\left(\Gamma_{i}=l|y_{i},\theta ,\Phi_{i}\right)} \end{array}$$

The previous equations are extended to multi-modal data as described in Van Leemput et al. (1999).

*Overview of Proposed Procedure with Enhancements:* This multi-modal MRI framework begins by taking inputs of any combination of modalities with any number of repetitions. Repeated scans within a single sessions can be taken advantage of to increase in signal-to-noise ratio for each modality. Our procedure begins with spatial normalization of each intra-modal scan into a common subject-specific reference orientation defined by the AC (Anterior commissure), PC (Posterior Commissure), and mid-sagittal plane by using Rigid-type transformation (Ghayoor et al., 2013). The spatial normalization reduces non-subject specific spatial variation between scans, and in turn, enhances robustness and efficiency of subsequent procedures. Subject-specific tissue posteriors are estimated by performing EM procedure described previously. The posterior estimation step here employs (1) atlas-to-subject high-deformable registration algorithm [ANTS (Avants et al., 2011a)] to enhance accuracy of subject specific tissue priors by increasing warping correspondence to the subject and (2) a novel region-specific intensity constraint to ensure the correctness of tissue posteriors. Finally, the bias-field of each input MR image is estimated and applied based on current estimate of tissues. The whole process iterates until converges: the step returns to posterior estimation with improved intensity homogeneity, consecutively brings upgraded estimation of inter-scan high deformable registration, and so improved tissue posterior estimation.

To accomplish superior robustness towards large-scale multi-site longitudinal MR data, each step is carefully reviewed, and tested (highlighted with gray in Figure 1): (1) Multi-modal images including their repetition images are incorporated to compensate a degraded MRI quality (Section 2.1), (2) high-degree of deformable registration is integrated for provide better estimate of spatial location of each tissue type (Section 2.2), (3) estimated tissue posteriors are constrained with intensity- context prior to accommodate shortcomings of atlas based tissue segmentation and/or bias correction (Section 2.3), and (4) 17 tissue priors (as opposed to three tissue priors) are employed to further stabilize the tissue classification algorithm and to address severe intra-scan bias (Section 2.4). The following section describes each enhancement in detail.

### 2.1. A collective set of input: multi-modal MR images with repetition

A collective set of multi-modal MR images including repetitions from a single scan session are utilized. It is well established that multi-modal MR images can provide complementary information that can improve brain tissue classification (Rubin, 1999). We further employed repeated scans, where they are available, to compensate inherent noise in the measurements. Careful study design and scanning protocols can limit the occurrence artifacts, but some are unavoidable.

### 2.2. Integrating high-deformable registration [SyN (Avants et al., 2011b)]

High deformable registration is integrated for accurate estimation of deformation mapping of atlas priors to subject subject specific space. We hypothesize that improved correspondence mapping of the atlas to the subject benefits tissue classification procedure as well as bias-field correction as compared to previously employed affine or B-Spline registrations. Symmetric image normalization (SyN) based registration (Avants et al., 2011b) provided from ANTS package is extensively tested and has been shown to perform well at preserving image topology. With the high deformable registration, our subject specific spatial priors are now further refined:

(4) $$p\left(\Gamma_{i}=l\right)=\frac{\psi \left(t_{il}\right)}{\sum_{j=1}^{K}\psi \left(t_{ij}\right)}$$

### 2.3. Extended prior definition

**Figure 2:**
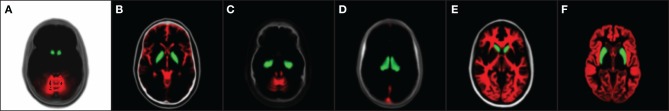
**17 Spatial priors**. *(Denote gray [gr], green [gn], and red [r])*. **(A)** Air [gr], accumben [gn], and cerebellum GM [r], **(B)** Not GM [gr], globus pallidus [gn], and CSF [r], **(C)** Not CSF [gr], hippocampus [gn], and cerebellum WM [r], **(D)** Not venous blood [gr], thalamus [gn], and venous blood [r], **(E)** Not WM [gr], caudate [gn], and cerebral WM [r], and **(F)** Putamen [gn] and surface cerebral GM [r].

Extended spatial tissue priors are employed for the robustness of large-scale MR data processing. Traditionally, spatial priors propagate tissue-specific spatial knowledge to a MR image-processing algorithm. One of the big assumptions behind utilization of tissue spatial priors is the homogeneous intensity profile of identical tissue type across images. In a large-scale study setting, however, the degree of inhomogeneity is vastly different from scan to scan. Rather than adjusting algorithmic parameterization for each problematic case, the pragmatic way to deal with the situation is to break down biological tissue definitions further by their unique image properties. We designed 17 extended tissue specific priors based on their spatial location, intensity profiles, and biological definitions (Figure 2). These priors are constructed to have intrinsic hierarchical tissue definitions with respect to each other (See Table 1 and Figure 2)

**Table 1:** **Atlas definition of 17 region-specific intensity-context prior**.

**Tissue**	**Name**	**Weight**	**Bias correction**	***q^T1^_lower_***	***q^T1^_upper_***	***q^T2^_lower_***	***q^T2^_upper_***
Gray matter	Accumben	1	False	0.05	0.95	0.15	0.97
	Caudate	1	False	0.05	0.95	0.15	0.97
	Crbl Gm	1	True	0.03	0.9	0.02	0.99
	Hippocampus	1	False	0.05	0.95	0.15	0.97
	Putamen	1	False	0.05	0.95	0.15	0.97
	Surf Gm	1	True	0.04	0.75	0.25	0.96
White matter	Wm	1	True	0.5	1	0.05	0.7
	Crbl Wm	1.5	True	0.1	1	0.03	0.9
Csf	Csf	1	True	0	0.6	0.2	1
Wm & Gm	Thalamus	1	False	0.05	0.95	0.15	0.97
	Globus	1	False	0.05	0.95	0.15	0.97
Venous blood	Vb	1	False	0.04	0.75	0	0.2
Background	Not Csf	1	False	0	0.6	0.2	1
	Not Gm	1	False	0.15	0.9	0.35	1
	Not Vb	1	False	0.15	0.9	0	0.3
	Not Wm	1	False	0.4	1	0.1	0.85
	Air	1	False	0	0.1	0	0.1

*Each Tissue type sub-divided into regions of interest with given Name*.

*Weight and whether it is used for bias correction computation are also shown*.

*Intensity-context prior definitions are their valid range according to quantized percentile of intensity, Quantiles (q^T1^_lower/upper_, q^T2^_lower/upper_), to take account MR's relative intensity and outliers. (Crbl = cerebellum)*.

The most prominent regions of interest include gray matter (GM), white matter (WM), and cerebrospinal fluid (CSF). Those prominent regions of interest are now partitioned further based on their spatial property, depending on whether it is located in cortical/subcortical (peripheral/central) of brain. The distinction of priors between cortical and subcortical tissues is practically useful since they often present heterogeneous MR intensity characteristics across different imaging modalities. In addition, the background region of image is also partitioned based on intensity profiles. Tissues in the background are named as ‘Not-tissue’ regions, which demonstrate similar MR-image profiles to the tissues of interest, but are spatially located outside of the brain/biological region of interest, such as bone, skin, or fat. Designation of each spatial tissue priors is summarized in Table 1.

### 2.4. Multi-modal region-specific intensity-context priors

Intensity-context priors are devised for algorithmic robustness of large-data processing. Since MR intensities are are not a standardized quantitative measurement, the threshold parameters for each tissue type are designated by quantiles of each image's histogram. The multi-modal quantile threshold value of each tissue type are conservatively chosen to ensure that each tissue regions is completely included (i.e., no false negatives). These threshold identified regions are used as an additional constraint in conjunction with their corresponding spatial priors. The set of multi-model threshold parameters that are used globally for our studies are shown in Table 1. By incorporating *a priori* knowledge β, multi-modal intensity constraints of each tissue type, the initial estimates of tissue statistics are made more robust across a wide range of imaging protocols. Therefore, for multi-modal intensity model *y* with a indicator function, defined **1**_β_(*y*_*il*_), then Equation 2 and Equation 4 can be further refined,

(5) $$\begin{array}{l} p(\Gamma_{i}=l)=\frac{\psi \left(t_{il}\right)\cdot {\bar{1}}_{\beta }\left({\bar{y}}_{il}\right)}{\sum_{j=1}^{K}\psi \left(t_{ij}\right)\cdot {\bar{1}}_{\beta }\left({\bar{y}}_{ij}\right)},\\ {\bar{1}}_{\beta }({\bar{y}}_{il})=\{\begin{array}{ll} 1, & \text{if }{\bar{y}}_{i}\in \beta \text{=}\{\bar{y}\text{|}q_{lower}^{m}<y^{m}<q_{upper}^{m}\text{for all image​ }m\text{​}\}\\ 0, & otherwise \end{array} \end{array}$$

The software implementation and usage is explained in Appendix A.

## 3. Evaluation

The accuracy and effectiveness of our proposed enhancements are evaluated from multiple perspectives: (1) compare similarity against known ground truth by using simulated MR data, (2) visual inspection results by experts, and (3) a visual comparison of sample results to the well-established techniques (FreeSurfer (Fischl et al., 2002; Reuter and Fischl, 2011) and Atropos from ANTS package (Avants et al., 2011a)).

### 3.1. Evaluation on simulated data

**Figure 3:**
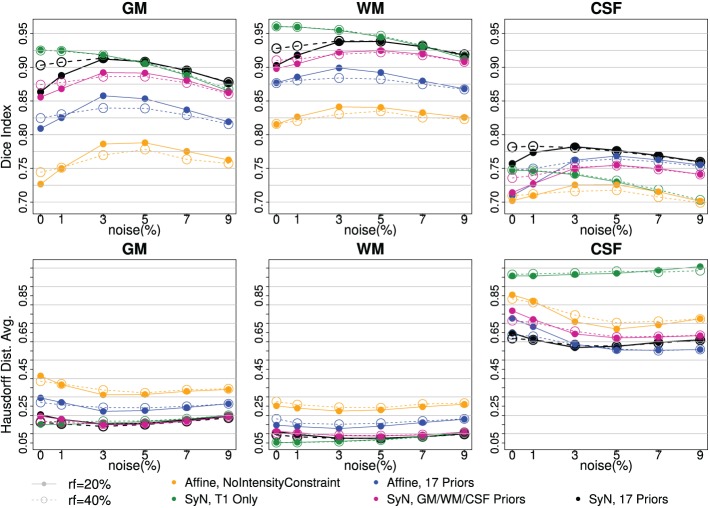
**Tissue classification agreement comparison to three-tissue based ground truth with BrainWeb Data**. Two bias-fields, *rf* = *20* (solid line) and *rf* = 40 (dotted line), are shown with six noise levels along x-axis. Two independent measures of Dice Index (upper) and average Hausdorff Distance (bottom) are shown. With affine registration, *Intensity-context prior* (yellow) has better accuracy than one without one (blue). *SyN* registration (pink, black) also improved tissue segmentation agreement further comparing to the *affine* (yellow, blue). Note that the performance with *SyN* registration utilizing only three tissue types outperformed any affine registration method. *Multi-modal input trial*, T1 and T2, (black) comparing to *T1 Only* (green) seems helpful when there is more noise.

A series of evaluation experiments with BrainWeb data are summarized in the Figure 3.

Two independent measures, Dice index (larger is better) and average Hausdorff distance (Dubuisson et al., 1994) (smaller is better), are reported to underscore the validity of our processing between the automated delineation and the ground truth. The visualization of results makes it clear there is a difference between with and without enhancements. Along the six noise levels with two degrees of bias-field, the most agreeable result to the ground truth was obtained by utilizing all of our proposed enhancements: high-deformable registration (SyN) with intensity-context priors (Figure 3:black).

The series of BRAINWeb experiments demonstrate the benefit of each individual enhancement, and also the combined benefit of using all enhancements together. First, high deformable registration (SyN) improves tissue classification results as compared to affine registration (blue vs. black). Second, multi-modal intensity constraints benefit the procedure, especially when registration is less optimal due to large morphological differences that often present in degenerative diseases (Figure 3: contrasting yellow vs. blue). Third, the extended definition of tissue priors helps to increase accuracy of segmentation (Figure 3:purple vs. black). Those three improvements were valid for all six levels of noise and two bias-field levels. Finally, using multi-modal input is beneficial especially when MR scan is corrupted with noise and/or inhomogeneity bias (Figure 3: black vs. green).

### 3.2. Evaluation on *in-vivo* data

The proposed pipeline was applied on the *in vivo* MR data, collected from the multi-site international PREDICT-HD (PREDICT-HD, 2001) project. The PREDICT-HD data (PREDICT-HD, 2001) was highly heterogeneous. The inhomogeneity of the data was due to a multi-site natural history observational study design that employed all the available resources including multiple MR vendors (GE, Phillips, and Siemens), field strengths (1.5*T* and 3*T*), and over 20 different MR acquisition protocols (i.e., due to transmission and receive hardware). All the processed images (*n* = 3751) are visually inspected by three independent experts and only <2% scans were classified as obvious failure to produce bias-corrected T1 images. Note that our proposed method achieved a higher process completion rate without error than FreeSurfer for the large-scale MR data collected at PREDICT-HD study. Processing time varied approximately between 3 and 5 h per scan (related to the number of modalities and repeats) for the entire bias correction, registration, and tissue classification.

**Figure 4:**
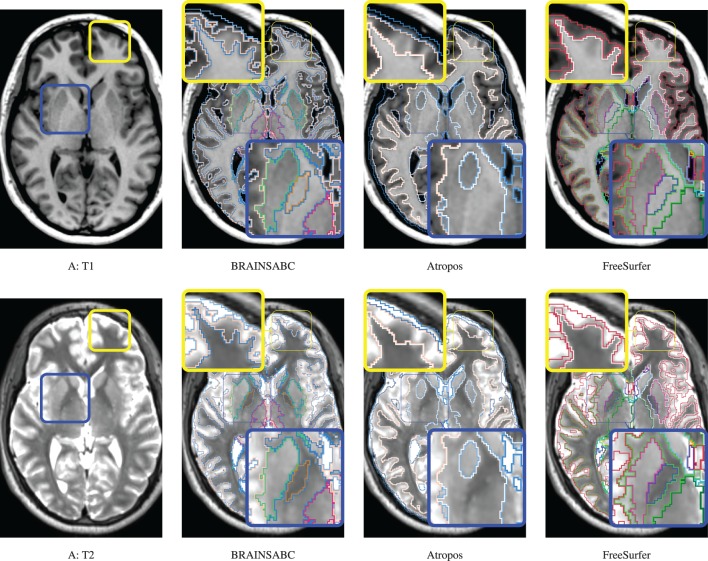
**Visual Comparison of Scan A in Table 2**. Tissue classification results from three applications, BRAINSABC, Atropos of ANTS Tools, and FreeSurfer on top of T1- and T2-weighted images, multi-modal repeated scan processing. This subject present relatively normal tissue ratio, reflecting minimal, if exists, atrophy in brain tissues. Red and blue boxes highlight where tissue classification is more differentiated from each other. BRAINSABC were highly agreeable to Atropos and FreeSurfer in cortical area in general. For subcortical area (blue box), however, BRAINSABC produced more agreeable to FreeSurfer than Atropos. Atropos were the most conservative in the subcortical GM identification among three methods. For CSF (or ventricle) region, which is more distinguishable from T2-weighted modality, BRAINSABC produced very robust results across scans.

**Figure 5:**
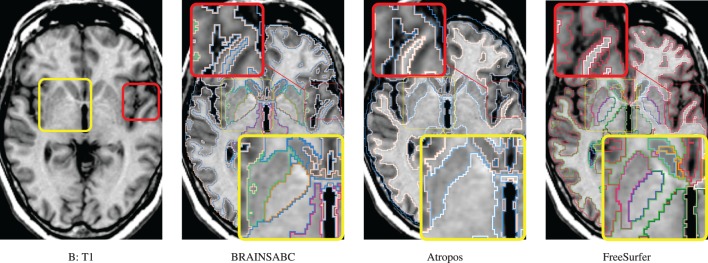
**Visual Comparison of Scan B in Table 2**. Tissue classification results from three applications, BRAINSABC, Atropos of ANTS Tools, and FreeSurfer on top of T1-weighted images, uni-modal processing. This subject present relatively small tissue ratio, reflecting brain atrophy progression to some extent. Again, Atropos were more parsimonious in the subcortical (yellow box) GM identification than others. Red box also underlines differences of tissue classifications on the cortical area. Note that without T2 modality, CSF classification results of BRAINSABC were more agreeable to the other two.

**Figure 6:**
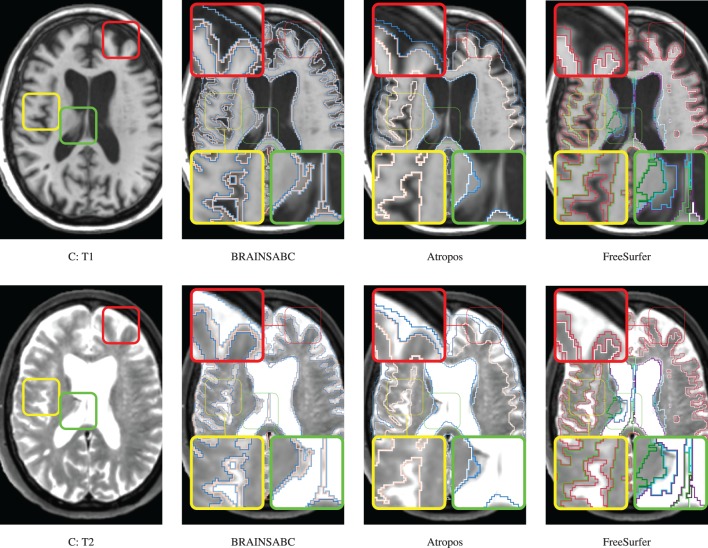
**Visual Comparison of Scan C in Table 2**. Tissue classification results from three applications, BRAINSABC, Atropos of ANTS Tools, and FreeSurfer on top of T1- and T2-weighted images, multi-modal processing. This subject present relatively small tissue ratio, reflecting brain atrophy progression to some extent. BRAINSABC and other two methods produced very similar results in this scan. Differences between methodologies are also observed as highlighted in red, yellow, and green boxes. Red box contrasts classification on the cortical region, where BRAINSCut draws a nice borderline for surface CSF in regarding to both T1- and T2-weighted images. Green box magnified CSF and GM border on the subject with enlarged ventricles where T1-weighted image shows artifacts. Last, yellow box also shows different classification results between tools. Again, CSF classification present some disagreement between FreeSurfer and BRAINSABC (green box).

Three subjects were sampled for a retrospective result comparison with regard to (1) MR vendors and (2) rough estimate of tissue ratio, volume of WM and GM over intracranial volume, to reflect morphological variations in our large-scale multi-site study. Characteristic of the sample data is summarized in Table 2. The smaller tissue ratio means more atrophy in brain tissue, which generally caused either by disease progression or aging. To facilitate visual comparison to other works, tissue classification results from Avants et al. (2011a) and Fischl et al. (2002); Reuter and Fischl (2011) are displayed as well. To be fair, multi-modal approaches (where applicable) are applied and their results are visually investigated for all three processing pipelines. In this study, we did not use *‘-T2’* option for FreeSurfer. As Figures 4, 5, 6 show, tissue boundaries in cortical area (peripheral region of brain) from our proposed approach (*BRAINSABC*) were more agreeable to other two methods than subcortical area (central area of brain). For the subcortical GM, however, three approaches resulted in noticeable differences. *BRAINSABC*'s results were closer to FreeSurfer while Atropos exhibited the most conservative subcortical GM delineation. Note that globus pallidium was treated as independent tissue type in BRAINSABC and FreeSurfer while there was no special consideration for globus pallidium in the Atropos.

**Table 2:** **Sample results are shown in this paper for visual comparison**.

**Scan**	**Site**	**MR Vendor**	**Field strength**	**Tissue ratio**	**Collected modality(#)**
A	Site_180	SIEMENS TrioTim	3T	>0.88	T1(2), T2(2)
B	Site_024	GE	1.5T	<0.78	T1(1)
C	Site_039	PHILIPS	3T	<0.78	T1(4), T2(2)

Processing results from three subjects that are collected at different sites with various characteristics in MR vendors, rough tissue ratios, field strengths, and number of repeats.

## 4. Discussion

*Summary:* We propose a method to advance the automatic bias-field correction algorithm for large-scale heterogeneous MR data processing. Our proposed method is evaluated via application to both simulated brain MR images as well as *in vivo* MRI collected from a large multi-center study. The series of experiments on simulated MR data revealed the improved robustness of our proposed enhancements in the presence of varying levels of noise, inhomogeneity. In addition, application to *in vivo* MRI, collected for multi-site study, also showed good generalizability as demonstrated by the very low failure rate across a wide spectrum of input image protocols. Sample results are also presented in comparison to the well-established tools of Atropos and FreeSurfer.

**Figure 7:**
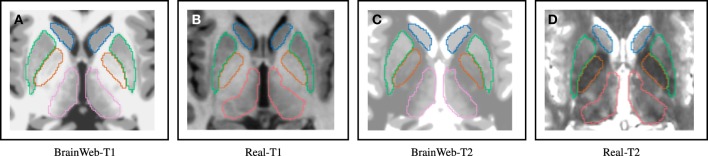
**Distinguishable intensity profile differences between BrainWeb and real MR data**. Difference is more obvious for the globus pallidus (orange) region, where BrainWeb data appears medium gray both in T1 and T2. Real data, however, presents the globus pallidus (orange) with medium gray in T1 and dark gray in T2. **(A)** Simulated T1; **(B)** *In-vivo* T1; **(C)** Simulated T2; **(D)** *In-vivo* T2.

With the evaluation with simulated data in Section 3.1, Dice index goes up at first and then down as the noise level increases. One possible explanation for the slight DSC increment as a bit of noise/bias added to a simulated MRI, is that a simulated MR image without noise/bias may be dissimilar to an *in vivo* MRI. A patient MRI *in vivo* is usually corrupted by noise to some extent. (Figure 7 shows differences between simulated and *in-vivo* MRIs). Since our techniques are highly optimized for *in vivo* MRIs, testing the method on simulated MR images without realistic noise corruption, may be a less than ideal validation.

The key contributions of this work are three fold: (1) pipeline enhancements for large-scale heterogeneous MR data processing, (2) empirically showing advantages of utilizing multiple scans including multi-modal or repeated MRIs, and (3) distributing all the tools of the open source pipeline including all parameter sets.

Additional advantages of our proposed enhancements are revealed from *in vivo* application. First, BRAINSABC requires no pre-alignment between scans because the process incorporates both intra-subject and atlas-to-subject registration with refinements in the iterative process. Second, as shown in Figure 6, the brain extraction of BRAINSABC produces very high quality brain region estimate as compared to other two approaches. A robust brain region estimation is important because it is often employed in normalizing sub-volumetric data to compensate for overall brain size differences between subjects. In addition, we found empirically that the visual inspection failure rate of FreeSurfer on the raw MRI scans was approximately 20%, on large-scale heterogeneous MR data, but when FreeSurfer was provided the BRAINSABC tissue classified pre-aligned and bias-corrected images the visual inspection failure rate dropped to approximately 8%.

A more comprehensive evaluation and validation of all available tissue classification tools to see how our proposed tool performs in comparison would have been ideal, but this task was determined beyond the scope of this paper. However, in this study, we have provided a formal validation study of our proposed tool as well as a formal comparative study against well-established tools. In addition, the results of our study has undergone a rigorous qualitative assessment that involved visual inspections by three independent experts who have been trained on a large number of scan sessions from various sites and scanning protocols.

The software implementation is written based on the *InsightToolkit* libraries and conforms to the coding style, testing, and software license guidelines specified by the National Alliance for Medical Image Computing group. Our implementation, BRAINSABC, is publicly available at https://github.com/BRAINSia/BRAINSTools via BRAINSTool package. Our implementation is optimized for, but not limited to, large-scale MR data analysis. The implementation has successfully applied over 3000 scans from the large-scale longitudinal study (PREDICT-HD, 2001) and visually inspected their validity. Advantages of our tool come primarily from its demonstrated generalizability to a wide number of scanning protocols, variations in the number and type of modalities, and number of repeated scans. As a part of larger image processing framework, this iterative automatic bias-field correction module provides very robust and consistent results for further MR image analysis.

## Funding

This research was supported by *NIH* Neurobiological Predictors of Huntington's Disease (PREDICT-HD; NS40068, NS050568) *National Alliance for Medical Image Computing* (NAMIC; EB005149 / Brigham and Women's Hospital), and *Enterprise Storage in a Collaborative Neuroimaging Environment* (S10 RR023392 / NCCR Shared Instrumentation Grant).

### Conflict of interest statement

The authors declare that the research was conducted in the absence of any commercial or financial relationships that could be construed as a potential conflict of interest.

## Appendix A: implementation and usage

The software implementation is written based on the *InsightToolkit* libraries and conforms with the coding style, testing, and software license guidelines specified by the National Alliance for Medical Image Computing group. Our implementation, *BRAINSABC*, is publicly available at https://github.com/BRAINSia/BRAINSTools via BRAINSTools package:

Once downloaded and built the BRAINSTools package, the standard help manual is available via –*help* command, or -*h* in short. Note that required inputs are written in blud bold letters.

Multi-modal repeated scan processing can be applied as following command: Note that the presented parameter set is used for entire this paper. Again, a set of required inputs are written in blue bold letters, and the file “ExtendedAtlasDefinition.xml” of –*atlasDefinition* is provided in the BRAINSTools package along with all the altases. The only adjustment we have made is the Affine and SyN of the –*atlasToSubjectTransformType* option, which can be chosen from five different options: Identity, Rigid, Affine, BSpline, and SyN
